# Global burden of tracheal, bronchus, and lung cancer attributable to second-hand smoke exposure from 1992 to 2021: an age-period-cohort analysis and 25-year mortality projections

**DOI:** 10.3389/fpubh.2025.1625876

**Published:** 2025-11-25

**Authors:** Hong-ming Lin, Bin Lin, Xin-peng Liao, Hong Hong, Xue-ting Cai, Xi-bin Zhuang

**Affiliations:** 1 Department of Respiratory and Critical Care Medicine, First Hospital of Quanzhou Affiliated to Fujian Medical University, Quanzhou, China; 2 Department of Gastric Surgery, Fujian Medical University Union Hospital, Fuzhou, China

**Keywords:** second-hand smoke (SHS), tracheal, bronchus, and lung (TBL) cancer, Global Burden of Disease(GBD), age-standardized mortality rate (ASMR), age-standardized DALY rate (ASDR), estimated annual percentage change (EAPC)

## Abstract

**Background:**

Tracheal, bronchus, and lung (TBL) cancer remains the leading cause of cancer-related mortality worldwide. While smoking is the primary risk factor, several non-smoking-related factors also significantly contribute to TBL cancer, notably second-hand smoke (SHS) exposure, which plays a substantial role in the disease’s burden. This study assesses and forecasts the temporal trends in the disease burden of TBL cancer attributable to SHS exposure at global, regional, and national levels.

**Methods:**

We extracted data from the ≥ 25 years population in the Global Burden of Disease Study 2021 (GBD 2021) to assess deaths, disability-adjusted life years (DALYs), age-standardized mortality rate (ASMR), and age-standardized DALY rate (ASDR) at both global and regional levels. We evaluated temporal trends using descriptive statistics, estimated annual percentage change (EAPC), and both the age-period-cohort (APC) and Bayesian age-period-cohort (BAPC) models.

**Results:**

From 1992 to 2021, absolute deaths and DALYs linked to TBL cancer due to SHS exposure increased, although ASMR and ASDR showed declining trends, with EAPC values of −0.92% and −1.30%, respectively. Regions with a high socio-demographic index (SDI) displayed the most significant improvements. In contrast, high-middle and middle SDI regions bore the greatest disease burden, and low SDI regions saw minimal progress. The highest ASMR in 2021 occurred mainly in Western Europe, North America, and East Asia, with ASMR correlating positively with SDI. The disease burden was consistently higher among males, particularly in the 65–74 age group, across both sexes. Future projections indicate a continuing decline in the disease burden from 2022 to 2046.

**Conclusion:**

SHS exposure continues to be a significant factor in the disease burden of TBL cancer. Although there has been an overall declining trend globally, it still caused the deaths of more than 97,000 people in 2021. There exists considerable heterogeneity among regions, with some areas still bearing a substantial disease burden. These findings highlight the need for specific prevention and control strategies to mitigate the health impacts of SHS exposure.

## 1 Introduction

Tracheal, bronchus, and lung (TBL) cancer is recognized as one of the most formidable malignancies worldwide and poses a significant challenge to public health. According to the International Agency for Research on Cancer (IARC), in 2022, approximately 2,480,675 cases of TBL cancer were documented, representing 12.4% of all cancer cases globally. Furthermore, TBL cancer accounted for 1,817,469 deaths, or 18.7% of all cancer-related fatalities, ranking it highest in both incidence and mortality among malignancies, with a trend that continues to rise (1). Projections indicate that by 2050, TBL cancer will account for 13.1% of new global cancer cases and 19.2% of cancer-related deaths (2), highlighting its escalating impact and persistent threat to public health.

Smoking remains the predominant risk factor for TBL cancer. Although there has been a significant reduction in smoking-related TBL cancer cases due to effective tobacco control measures over recent decades, some studies report an increase in cases among never-smokers (3). Non-smoking risk factors contributing to TBL cancer include airborne particulate matter pollution, occupational exposures (such as asbestos), and second-hand smoke (SHS) exposure. SHS, also known as passive smoking or environmental tobacco smoke, refers to the inhalation of tobacco smoke by non-smokers, with sidestream smoke being the primary component of SHS, containing multiple harmful substances similar to those found in active smoking (4). Current research demonstrates that SHS is highly correlated with increased risk of cardiovascular disease (5), respiratory diseases (6), and TBL cancer (7). In 2019, the World Health Organization (WHO) estimated that SHS resulted in a global loss of 37 million DALYs and caused 1.3 million deaths (8). It is estimated that 37% of the global population remains exposed to smoke emitted from the burning end of tobacco products or exhaled by smokers (9, 10). The IARC has now classified SHS as a Group 1 carcinogen, confirming its established role in causing human cancers, particularly TBL cancer (11). Research has identified over 7,000 chemicals in SHS, including at least 80 recognized carcinogens (12). These substances initiate the malignant transformation of lung cells through various mechanisms such as genetic mutations, epigenetic alterations, and oxidative stress (13–15). The impact of SHS exposure on TBL cancer risk has gained significant attention in recent years. Studies have shown that non-smokers with long-term exposure to SHS have a 20–30% increased risk of developing TBL cancer (16, 17). A meta-analysis on the association between SHS and TBL cancer revealed that never-smokers exposed to SHS have a significantly higher risk of cancer compared to those unexposed, with a relative risk (RR) of 1.24 (95% CI: 1.16 to 1.32) (18). These epidemiological studies further validate the link between SHS exposure and the development of TBL cancer.

Given the significant disease burden of TBL cancer on public health, although numerous studies have explored the association of TBL cancer with smoking, outdoor particulate pollution, and occupational hazards (19–23), there are no published papers that describe and explore the long-term burden trends of TBL cancer caused by SHS categorized by age, gender, location, and SDI using APC and BAPC models. Therefore, this study aims to utilize APC and BAPC models to quantify and predict the TBL cancer burden attributable to SHS exposure in 204 countries and territories from 1992 to 2021, stratified by age, sex, location, and SDI. This approach not only separates the effects of aging, historical factors of risk exposure, and vulnerabilities specific to birth cohorts, but also projects the age-standardized mortality rates for secondhand smoke-attributable lung cancer from 2022 to 2046. Through this comprehensive analysis, we aim to provide robust and detailed scientific evidence to elucidate the long-term effects of SHS on TBL cancer, offer key insights for the development of targeted intervention strategies, and ultimately drive global advancements in TBL cancer prevention and control.

## 2 Materials and methods

### 2.1 Study data

The GBD study, initiated in 1990, offers a foundational data source for global health research through its comprehensive scope and systematic methodology. This international collaborative project covers 204 countries and territories, evaluating 371 diseases and injuries alongside 88 risk factors. These locations are further categorized into 21 regions and 7 super-regions (24). In our study, we utilized secondary data from the GBD collaborative platform to assess the burden of TBL cancer attributable to SHS exposure. We analyzed relevant data at global, national, and regional levels for 2021 and projected trends from 2022 to 2046.

The codes for TBL cancer were sourced from the International Classification of Diseases (ICD)-9 and ICD-10 coding books. The ICD-9 codes include 162-162.9, 209.21, V10.1-V10.20, V16.1-V16.2, and V16.4-V16.40, while ICD-10 codes include C33, C34-C34.92, Z12.2, Z80.1-Z80.2, and Z85.1-Z85.20 (25). SHS exposure is defined as the inhalation of tobacco smoke in household, occupational, or public environments (26). Regarding the attribution estimation methods for TBL cancer attributable to SHS exposure, GBD 2021 followed the general framework established by Comparative Risk Assessment (CRA), which can be divided into six key steps: identifying SHS-TBL cancer pairs based on systematic reviews and meta-regressions; estimating relative risks from SHS-TBL cancer risk curves using the meta-regressions, regularized, and trimmed (MR-BRT) method; estimating the age-sex-location-year distribution of ambient SHS exposure levels; setting the theoretical minimum risk exposure level (TMREL) as zero exposure, meaning complete avoidance of SHS exposure; considering different exposure scenarios in household environments, workplaces, and public places, using spatiotemporal modeling methods to estimate exposure distributions in data-sparse regions; and estimating population attributable fractions and attributable burden using formulas that account for risk functions, individual exposure distributions across age-sex-location-years, and TMREL, while quantifying uncertainty intervals of estimates through 1,000 uncertainty draws (8, 25, 27). Notably, the GBD 2021 database excludes data on SHS-attributable TBL cancer burden for populations under 25 years old; hence, our study focuses on those aged 25 and older. Through the GBD Results Tool^1^, we extracted annual data on SHS-attributable TBL cancer deaths, disability-adjusted life years (DALYs), age-standardized mortality rate (ASMR), and age-standardized DALY rate (ASDR) from 1992 to 2021, stratified by sex, age, and geographic location. The GBD database includes 21 GBD regions and 204 countries or territories, which are categorized into five SDI quintiles: low, low-middle, middle, high-middle, and high. The SDI is a composite development indicator comprising total fertility rate, average educational attainment, and per capita disposable income. It ranges from 0 (theoretical minimum development) to 1 (theoretical maximum development), reflecting a country’s overall social and economic development. Moreover, this study uses 15 age groups (in 5-year intervals from 25 to 94 years and ≥95 years), providing a multidimensional, detailed framework for evaluating the global burden of TBL cancer attributable to SHS exposure.

### 2.2 Statistical analysis

To examine the burden of TBL cancer attributable to SHS exposure, we implemented a multi-level statistical analysis. We used the Estimated Annual Percentage Change (EAPC) to quantify long-term trends in the ASMR and ASDR rate from 1992 to 2021. We applied a log-linear regression model:

ln(y)=α+βx+ε

where y represents ASMR or ASDR, and x denotes the year. The EAPC and its 95% CI were calculated as:

100×(exp(β)−1)

Temporal trends were categorized as increasing if the 95% CI of the EAPC estimate exceeded 0, decreasing if the 95% CI was below 0, and stable if the 95% CI included 0.

We evaluated the associations between ASMR/ASDR and the SDI using Pearson correlation and linear regression analyses, with statistical significance determined by two-tailed *p*-values < 0.05.

The age-period-cohort (APC) model, a cornerstone in contemporary epidemiological research, disentangles epidemiological indicators into three temporal dimensions: age, period, and birth cohort. Age effects describe the impact of biological aging on disease occurrence, period effects capture the impact of specific historical periods on disease incidence, and cohort effects reflect variations in risk factor exposure across different birth cohorts (28). We divided the study population into 15 consecutive 5-year age groups (from 25 to 99 years) during the period from 1992 to 2021, covering 20 consecutive birth cohorts. We analyzed key parameters, using the APC Web Tool provided by the National Cancer Institute. Parameter significance was assessed with Wald chi-square tests.

Furthermore, we employed the Bayesian age-period-cohort (BAPC) model with integrated nested Laplace approximations (using R packages BAPC and INLA) to project ASMR for TBL cancer attributable to SHS exposure from 2022 to 2046 (29). The ‘BAPC ()’ and ‘INLA ()’ functions were employed to fit the age-period-cohort model and estimate posterior distributions of model parameters, respectively. Data were structured into 5-year age intervals (specific age groups were categorized as under 5 years, 5–9 years, 10–14 years, etc., up to 95 years and above, totaling 20 age groups), with a time span from 1990 to 2021, incrementing annually. Population data and disease rates for each age group and period were prepared in matrix format, with rows representing age groups and columns representing periods. These data were input into the ‘BAPC ()’ function, with age and time dimensions specified accordingly. Standard population data were sourced from the GBD Study 2021, available at [IHME GBD 2021 Demographics] (Global Burden of Disease Study 2021 (GBD 2021) Demographics 1950-2021|GHDx). This dataset provides age-specific population weights from 1950 to 2021, which were used for standardized analysis in this study. The 95% uncertainty intervals (UIs) for predicted rates were calculated using posterior distributions derived from the BAPC model. These data were obtained through the ‘INLA’ package, which provides posterior marginal distributions for each parameter. The uncertainty intervals were calculated as the 2.5th and 97.5th percentiles of the posterior distributions (29). Compared to other forecasting methods, this approach not only provides superior prediction coverage and accuracy, but also offers important scientific evidence for future public health policy development (30). All statistical analyses were conducted in R software (version 4.4.1), ensuring methodological consistency and reproducibility.

## 3 Results

### 3.1 TBL cancer burden attributable to SHS exposure by global, SDI and GBD regions

The global burden of TBL cancer attributable to SHS exposure has shown significant changes from 1992 to 2021. The number of global deaths increased from 59,998.3 (95% Uncertainty Interval [UI]: 7,076.9 to 113,306.0) in 1992 to 97,910.8 (95% UI: 11,955.2 to 184,912.9) in 2021. Despite the rise in absolute numbers, the ASMR decreased from 1.44 (95% UI: 0.17 to 2.73) to 1.14 (95% UI: 0.14 to 2.15) per 100,000 population, with an EAPC of −0.92 (95% CI: −0.98 to −0.87). DALYs increased from 1,654,275.7 (95% UI: 195,097.3 to 3,113,584.6) to 2,355,866.0 (95% UI: 290,210.9 to 4,442,996.3), while the ASDR decreased from 38.09 (95% UI: 4.49 to 71.66) to 26.93 (95% UI: 3.32 to 50.83) per 100,000 population, with an EAPC of −1.30 (95% CI: −1.35 to −1.25) (Table 1).

**Table 1:** Global, SDI, and GBD regional burden of TBL cancer attributable to SHS exposure in 1992 and 2021, and the temporal trends from 1992 to 2021.

Location	1992				2021				EAPC in ASMR (*N*, 95%CI)	EAPC in ASDR (*N*, 95%CI)
	Deaths number (*N*, 95%UI)	ASMR (N,95%UI)	DALYS number (*N*, 95%UI)	ASDR (*N*, 95%UI)	Deaths number (*N*, 95%UI)	ASMR (*N*, 95%UI)	DALYS number (*N*, 95%UI)	ASDR (*N*, 95%UI)		
Global	59998.3 (7076.9 to 113,306)	1.44 (0.17 to 2.73)	1654275.7 (195097.3 to 3113584.6)	38.09 (4.49 to 71.66)	97910.8 (11955.2 to 184912.9)	1.14 (0.14 to 2.15)	2355866.0 (290210.9 to 4442996.3)	26.93 (3.32 to 50.83)	−0.92 (−0.98 to −0.87)	−1.30 (−1.35 to −1.25)
Sex										
Male	37714.1 (4307.6 to 71211.6)	1.99 (0.23 to 3.76)	1041110.6 (119615.1 to 1957408.4)	50.65 (5.79 to 95.37)	56848.1 (6655.0 to 109070.9)	1.44 (0.17 to 2.78)	1359556.6 (158,640 to 2596453.4)	32.88 (3.85 to 62.82)	−1.11 (−1.19 to −1.03)	−1.51 (−1.58 to −1.44)
Female	22284.2 (2779.1 to 41282.4)	1.00 (0.12 to 1.86)	613165.1 (75904.9 to 1129960.3)	27.02 (3.34 to 49.81)	41062.7 (5535.9 to 78059.2)	0.89 (0.12 to 1.69)	996309.4 (135988.2 to 1,907,079)	21.81 (2.98 to 41.75)	−0.65 (−0.71 to −0.58)	−0.97 (−1.03 to −0.91)
SDI region										
High SDI	19013.4 (2296.6 to 36483.3)	1.69 (0.2 to 3.24)	505916.9 (61216.0 to 964143.4)	46.47 (5.62 to 88.44)	16945.3 (2216.0 to 32742.6)	0.82 (0.11 to 1.58)	380284.8 (49959.2 to 738239.8)	19.99 (2.63 to 38.76)	−2.64 (−2.75 to −2.54)	−3.01 (−3.12 to −2.9)
High-middle SDI	22746.5 (2607.7 to 43436.4)	2.17 (0.25 to 4.13)	634725.4 (73284.5 to 1208733.5)	58.81 (6.79 to 112.04)	39124.1 (4613.2 to 73341.1)	1.96 (0.23 to 3.67)	936576.8 (111577.1 to 1736627.3)	47.20 (5.65 to 87.54)	−0.42 (−0.53 to −0.32)	−0.86 (−0.97 to −0.75)
Middle SDI	15272.3 (1820.9 to 28807.7)	1.44(0.17 to 2.68)	428644.3 (51160.3 to 805871.4)	36.25 (4.31 to 68.19)	35511.4 (4496.6 to 67456.6)	1.36 (0.17 to 2.58)	862344.5 (110217.8 to 1626034.8)	31.13 (3.97 to 58.93)	−0.28 (−0.34 to −0.22)	−0.61 (−0.67 to −0.56)
Low-middle SDI	2484.8 (324.3 to 4669.2)	0.39 (0.05 to 0.74)	71149.1 (9124.2 to 133912.5)	10.19 (1.32 to 19.15)	5477.7 (698.6 to 10459.2)	0.38 (0.05 to 0.73)	152291.8 (19443.6 to 291023.5)	9.90 (1.26 to 18.89)	−0.16 (−0.20 to −0.12)	−0.16 (−0.20 to −0.12)
Low SDI	394.2 (44.2 to 778.8)	0.17 (0.02 to 0.34)	11388.3 (1260.9 to 22404.4)	4.41 (0.49 to 8.69)	769.7 (93.9 to 1556.2)	0.15 (0.02 to 0.31)	22326.6 (2712.2 to 45067.7)	3.96 (0.48 to 8.01)	−0.51 (−0.58 to −0.43)	−0.56 (−0.64 to −0.49)
GBD region										
Central Asia	671.4 (85 to 1323.3)	1.34 (0.17 to 2.63)	20122.6 (2535.2 to 39914.2)	38.61 (4.86 to 76.34)	600.2 (77.9 to 1192.1)	0.73 (0.09 to 1.44)	16793.3 (2175.0 to 33398.2)	18.75 (2.44 to 37.2)	−1.69 (−1.83 to −1.55)	−2.15 (−2.27 to −2.04)
Central Europe	3,878 (488.6 to 7414.4)	2.51 (0.32 to 4.79)	112383.9 (14109.7 to 214408.1)	72.93 (9.14 to 138.96)	3494.4 (437.6 to 6728.9)	1.61 (0.20 to 3.1)	86225.3 (10669.4 to 165607.0)	42.31 (5.22 to 81.04)	−1.57 (−1.76 to −1.38)	−1.93 (−2.14 to −1.72)
Eastern Europe	4329.0 (555.2 to 8371.3)	1.47 (0.19 to 2.83)	125198.6 (15825.0 to 242080.3)	42.70 (5.36 to 82.29)	2826.5 (353.9 to 5554.2)	0.80 (0.10 to 1.58)	75436.3 (9497.2 to 147002.9)	22.23 (2.83 to 43.42)	−2.22 (−2.37 to −2.06)	−2.43 (−2.58 to −2.27)
Australasia	274.0 (27.3 to 577.0)	1.13 (0.11 to 2.37)	7225.9 (736.6 to 15020.2)	30.58 (3.13 to 63.91)	242.0 (25.7 to 525.7)	0.47 (0.05 to 1.01)	5814.2 (611.4 to 12570.5)	12.12 (1.27 to 26.13)	−3.06 (−3.16 to −2.95)	−3.19 (−3.28 to −3.1)
High-income Asia Pacific	2843.0 (371.9 to 5433.7)	1.33 (0.17 to 2.55)	70058.1 (9149.5 to 132926.9)	32.14 (4.20 to 61.00)	3440.5 (436.3 to 6957.1)	0.68 (0.09 to 1.4)	62672.9 (7815.6 to 128139.2)	14.83 (1.86 to 30.46)	−2.59 (−2.79 to −2.39)	−2.91 (−3.12 to −2.7)
High-income North America	6981.2 (840.1 to 13552.8)	2.02 (0.24 to 3.91)	185110.3 (22299.2 to 357286.6)	56.10 (6.74 to 107.75)	4567.5 (588.5 to 9059.5)	0.70 (0.09 to 1.38)	107412.8 (13712.8 to 211496.2)	17.36 (2.21 to 34.11)	−3.87 (−4.01 to −3.73)	−4.22 (−4.36 to −4.09)
Southern Latin America	713.8 (88 to 1460.2)	1.48 (0.18 to 3.03)	19911.0 (2493.7 to 41236.5)	41.17 (5.16 to 85.28)	614.2 (73.6 to 1313.5)	0.71 (0.08 to 1.52)	15436.2 (1844.6 to 33085.5)	18.30 (2.18 to 39.20)	−2.45 (−2.67 to −2.24)	−2.72 (−2.96 to −2.48)
Western Europe	8854.8 (1029.8 to 16,868)	1.58 (0.18 to 3.01)	239150.6 (27966.6 to 455458.0)	45.05 (5.29 to 85.78)	6226.9 (775.5 to 12307.0)	0.73 (0.09 to 1.43)	150798.7 (19175.0 to 295048.8)	19.46 (2.49 to 38.01)	−2.64 (−2.73 to −2.54)	−2.85 (−2.97 to −2.73)
Andean Latin America	50.0 (6.4 to 95.2)	0.23 (0.03 to 0.44)	1377.0 (177.6 to 2611.3)	5.92 (0.76 to 11.22)	67.3 (8.0 to 131.9)	0.11 (0.01 to 0.22)	1725.6 (200.5 to 3399.8)	2.84 (0.33 to 5.59)	−2.96 (−3.27 to −2.65)	−3.05 (−3.38 to −2.72)
Caribbean	274.9 (33.5 to 552.2)	1.03 (0.13 to 2.07)	6464.6 (797.9 to 12826.7)	23.50 (2.90 to 46.67)	356.8 (37.7 to 726.8)	0.66 (0.07 to 1.34)	8086.3 (863.4 to 16,491)	14.96 (1.60 to 30.52)	−1.62 (−1.81 to −1.44)	−1.69 (−1.88 to −1.49)
Central Latin America	350.9 (42.6 to 653.2)	0.41 (0.05 to 0.77)	9135.0 (1107.3 to 17006.9)	9.80 (1.19 to 18.22)	455.4 (54.9 to 865.9)	0.18 (0.02 to 0.35)	11229.6 (1346.2 to 21276.9)	4.39 (0.53 to 8.31)	−3.02 (−3.15 to −2.89)	−3.03 (−3.17 to −2.89)
Tropical Latin America	961.4 (116.2 to 1863.1)	1.00 (0.12 to 1.94)	26457.3 (3206.6 to 51031.4)	25.52 (3.09 to 49.32)	1368.4 (161.1 to 2745.1)	0.53 (0.06 to 1.07)	34367.8 (4092.8 to 69073.7)	13.08 (1.56 to 26.27)	−2.36 (−2.46 to −2.25)	−2.51 (−2.60 to −2.42)
North Africa and Middle East	2285.2 (240.2 to 4314.0)	1.31 (0.14 to 2.46)	63963.3 (6726.1 to 121560.1)	33.16 (3.47 to 62.62)	4533.8 (526.2 to 8819.1)	1.04 (0.12 to 2.03)	120068.9 (13861.4 to 232534.4)	24.83 (2.88 to 48.46)	−0.76 (−0.89 to −0.63)	−0.99 (−1.11 to −0.88)
South Asia	1632.9 (209.1 to 3071.6)	0.27 (0.03 to 0.52)	47044.2 (6057.3 to 88450.3)	7.06 (0.91 to 13.28)	3868.7 (526.3 to 7497.1)	0.26 (0.04 to 0.51)	108253.3 (14692.2 to 210037.6)	6.85 (0.93 to 13.28)	−0.56 (−0.73 to −0.40)	−0.48 (−0.64 to −0.32)
East Asia	23169.0 (2713.0 to 43997.0)	2.67 (0.31 to 5.06)	643834.9 (76237.1 to 1224395.0)	65.77 (7.75 to 125.39)	59196.3 (7267.3 to 111539.5)	2.75 (0.34 to 5.18)	1387974.7 (172610.9 to 2594495.8)	62.42 (7.79 to 116.40)	0.06 (−0.07 to 0.18)	−0.26 (−0.36 to −0.16)
Oceania	18.9 (2.2 to 39.7)	0.68 (0.08 to 1.42)	520.0 (59.3 to 1084.0)	16.09 (1.83 to 33.72)	51.1 (6.5 to 112.7)	0.75 (0.10 to 1.66)	1411.1 (178.9 to 3141.3)	17.75 (2.26 to 39.22)	0.29 (0.21 to 0.38)	0.29 (0.20 to 0.38)
Southeast Asia	2279.4 (316.1 to 4336.9)	0.87 (0.12 to 1.67)	63490.0 (8906.3 to 120403.6)	21.90 (3.05 to 41.56)	5325.6 (696.1 to 10098.1)	0.83 (0.11 to 1.57)	142236.7 (18518.9 to 271985.8)	20.32 (2.66 to 38.74)	−0.40 (−0.48 to −0.31)	−0.47 (−0.55 to −0.39)
Central Sub-Saharan Africa	38.9 (4.7 to 82.8)	0.16 (0.02 to 0.33)	1192.0 (144.0 to 2541.7)	4.36 (0.52 to 9.31)	84.4 (9.5 to 179.9)	0.14 (0.02 to 0.31)	2670.5 (296.9 to 5690.7)	3.99 (0.45 to 8.51)	−0.29 (−0.62 to 0.05)	−0.27 (−0.60 to 0.06)
Eastern Sub-Saharan Africa	92.9 (11.6 to 185)	0.12 (0.01 to 0.23)	2806.6 (349.8 to 5589.4)	3.19 (0.40 to 6.35)	142.0 (17.5 to 272.2)	0.08 (0.01 to 0.16)	4306.2 (533.1 to 8218.4)	2.21 (0.27 to 4.22)	−1.35 (−1.47 to −1.24)	−1.48 (−1.6 to −1.36)
Southern Sub-Saharan Africa	230.8 (28.7 to 437.3)	0.79 (0.1 to 1.5)	6949.1 (862.9 to 13133.0)	21.92 (2.72 to 41.38)	306.6 (39.7 to 584.2)	0.52 (0.07 to 0.99)	8950.4 (1159.7 to 17,074)	14.07 (1.82 to 26.85)	−1.57 (−1.8 to −1.33)	−1.64 (−1.87 to −1.40)
Western Sub-Saharan Africa	68.0 (8.6 to 133.0)	0.08 (0.01 to 0.15)	1880.8 (238.5 to 3657.0)	1.92 (0.24 to 3.74)	142.1 (17.7 to 277.6)	0.07 (0.01 to 0.15)	3994.9 (491.5 to 7784.4)	1.85 (0.23 to 3.61)	−0.1 (−0.18 to −0.02)	−0.21 (−0.29 to −0.13)

Significant disparities in the burden and trends of TBL cancer were evident across SDI regions in 2021. High-middle SDI regions experienced the highest burden with 39,124.1 deaths (95% UI: 4,613.2 to 73,341.1), followed by middle SDI regions with 35,511.4 deaths (95% UI: 4,496.6 to 67,456.6). In contrast, low SDI regions had the lowest burden with 769.7 deaths (95% UI: 93.9 to 1,556.2) (Table 1). High SDI regions demonstrated the most significant improvements in both the ASMR (EAPC: −2.64 [95% CI: −2.75 to −2.54]) and ASDR (EAPC: −3.01 [95% CI: −3.12 to −2.90]), with deaths decreasing to 16,945.3 (95% UI: 2,216.0 to 32,742.6) and DALYs declining to 380,284.8 (95% UI: 49,959.2 to 738,239.8) (Table 1). Conversely, both high-middle and middle SDI regions showed increases in absolute deaths and DALYs, despite moderate decreases in age-standardized rates.

Among GBD regions in 2021, East Asia, Western Europe, and High-income North America bore substantial burdens. East Asia faced the highest burden with 59,196.3 deaths (95% UI: 7,267.3 to 111,539.5) and 1,387,974.7 DALYs (95% UI: 172,610.9 to 2,594,495.8), showing minimal improvement in ASMR (EAPC: 0.06 [95% CI: −0.07 to 0.18]) (Table 1). Western Europe ranked second with 6,226.9 deaths (95% UI: 775.5 to 12,307.0) and 150,798.7 DALYs (95% UI: 19,175.0 to 295,048.8), followed by High-income North America, which demonstrated the largest improvements in both ASMR (EAPC: −3.87 [95% CI: −4.01 to −3.73]) and ASDR (EAPC: −4.22 [95% CI: −4.36 to −4.09]). Western Europe also showed notable improvements (EAPC in ASMR: −2.64 [95% CI: −2.73 to −2.54]; EAPC in ASDR: −2.85 [95% CI: −2.97 to −2.73]), while Oceania notably exhibited an upward trend in ASMR (EAPC: 0.29 [95% CI: 0.21 to 0.38]) (Table 1).

### 3.2 TBL cancer burden attributable to SHS exposure by national regions

**Figure 1:**
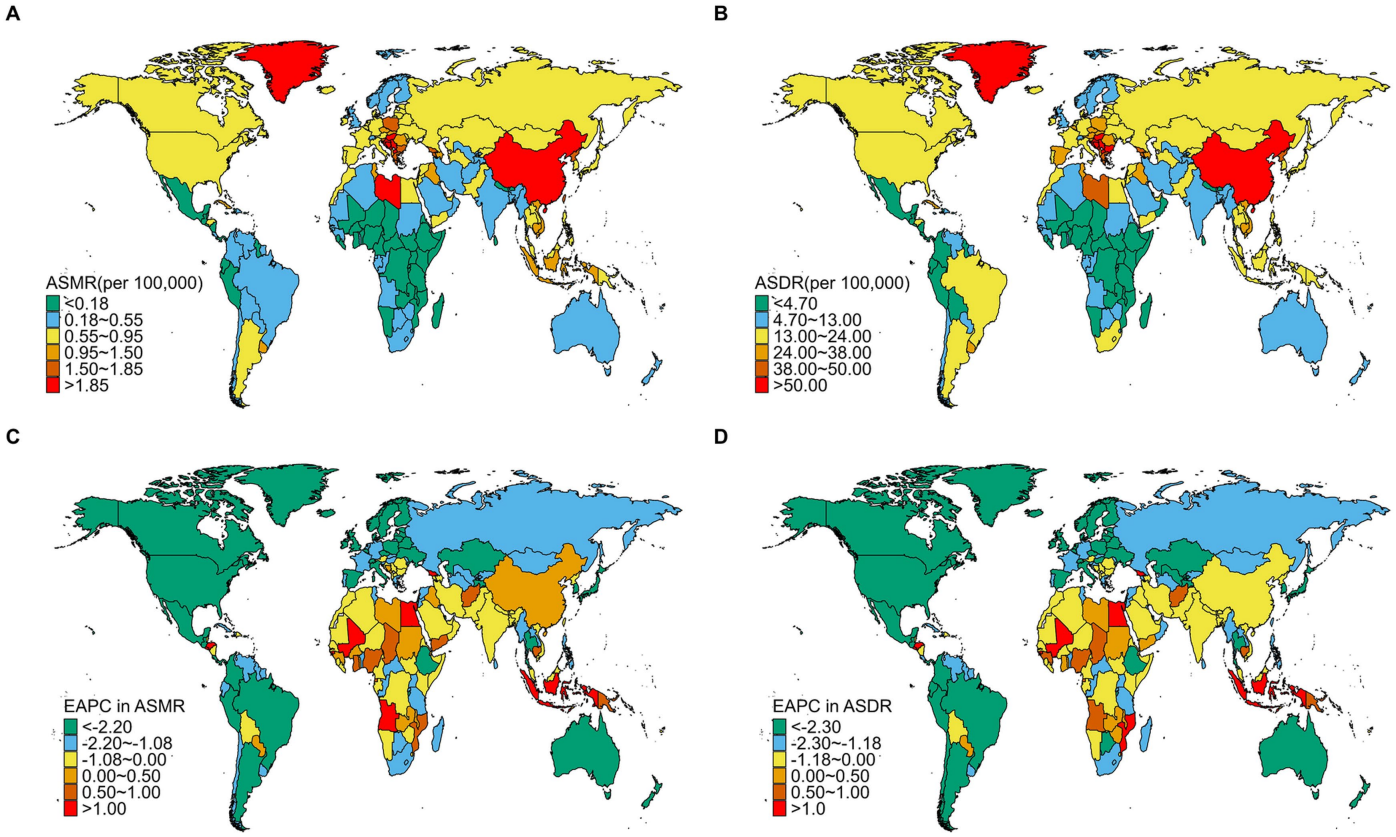
The spatial distribution of TBL cancer ASMR **(A)** and ASDR **(B)** attributable to SHS exposure in 2021, and the EAPC in TBL cancer ASMR **(C)** and ASDR **(D)** attributable to SHS exposure from1992 to 2021.

In 2021, significant disparities in the disease burden of TBL cancer attributable to SHS exposure were observed across national regions. The highest ASMR were recorded in Montenegro (3.45, 95% UI: 0.38 to 7.06), China (2.80, 95% UI: 0.35 to 5.27), and North Macedonia (2.55, 95% UI: 0.29 to 5.31) (Supplementary Table S1; Figure 1A). A similar distribution pattern was seen in the ASDR rates, with Montenegro (84.34, 95% UI: 9.36 to 170.96), China (63.32, 95% UI: 7.95 to 117.85), and North Macedonia (63.26, 95% UI: 7.03 to 132.65) reporting the highest burdens (Supplementary Table S1; Figure 1B). Conversely, the lowest burdens were predominantly observed in African regions, with Nigeria (0.02, 95% UI: 0.00 to 0.04), Kenya (0.04, 95% UI: 0.01 to 0.09), and Burundi (0.05, 95% UI: 0.01 to 0.11) reporting the lowest rates (Supplementary Table S1; Figure 1A). Between 1992 and 2021, most countries showed declining trends in both ASMR and ASDR rates, mainly in Western Europe and North America, such as the United Kingdom, Ireland, Denmark, Switzerland, the United States, Canada and Mexico (Supplementary Table S3; Supplementary Figure S2). In contrast, a few countries showed an upward trend mainly in Africa, such as Egypt, Lesotho, Guinea-Bissau, Mali, Mozambique, Angola, Georgia (Supplementary Table S3; Supplementary Figure S2). Developed nations typically demonstrated sustained improvements, whereas certain developing regions faced deteriorating conditions. Notably, some African countries consistently reported the lowest disease burdens, exemplified by Nigeria and Burundi. This pattern likely reflects limitations in disease surveillance and healthcare system capacities rather than genuinely lower disease burdens.

### 3.3 TBL cancer burden attributable to SHS exposure by sexes

**Figure 2:**
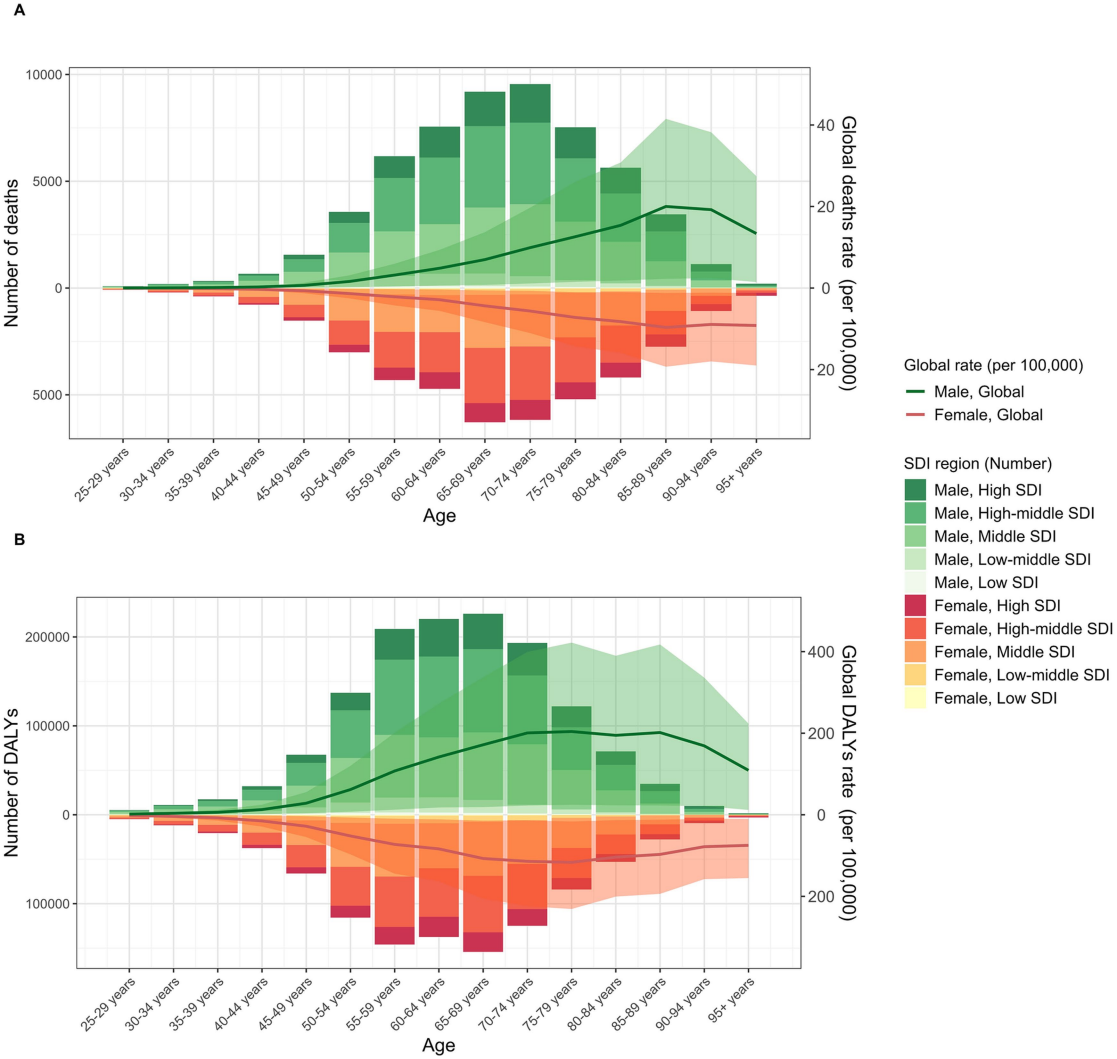
The burden of TBL cancer deaths **(A)** and DALYs **(B)** attributable to SHS exposure. The bars represent the number of TBL deaths and DALYs attributable to SHS exposure. The line with 95% UI represents ASMR and ASDR attributable to SHS exposure.

The disease burden of TBL cancer attributable to SHS exposure also shows significant differences between genders. Across both sexes, males consistently experienced a higher burden, with 56,848.1 deaths (95% UI: 6,655.0 to 109,070.9) and an ASMR of 1.44 (95% UI: 0.17 to 2.78) in 2021, compared to 41,062.7 deaths (95% UI: 5,535.9 to 78,059.2) and an ASMR of 0.89 (95% UI: 0.12 to 1.69) in females. Likewise, DALYs for males rose to 1,359,556.6 (95% UI: 158,640 to 2,596,453.4) with an ASDR of 32.88 (95% UI: 3.85 to 62.82), while DALYs for females reached 996,309.4 (95% UI: 135,988.2 to 1,907,079) with an ASDR of 21.81 (95% UI: 2.98 to 41.75) (Table 1). However, males showed greater improvement in age-standardized rates compared to females (EAPC in ASMR: −1.11 [95% CI: −1.19 to −1.03]; EAPC in ASDR: −1.51 [95% CI: −1.58 to −1.44]) (Table 1). Notably, TBL cancer deaths attributable to SHS exposure for both sexes followed a similar age pattern, increasing with age before gradually decreasing, predominantly concentrated in the 65–74 age group (Figure 2A). The burden of DALYs showed similar age and sex distribution patterns but peaked earlier at ages 65–69, with the primary burden concentrated in the 55–69 age group (Figure 2B). Notably, the disease burden was consistently higher in males than in females, a pattern that persisted across both developed and developing countries.

### 3.4 TBL cancer burden attributable to SHS exposure by SDI

**Figure 3:**
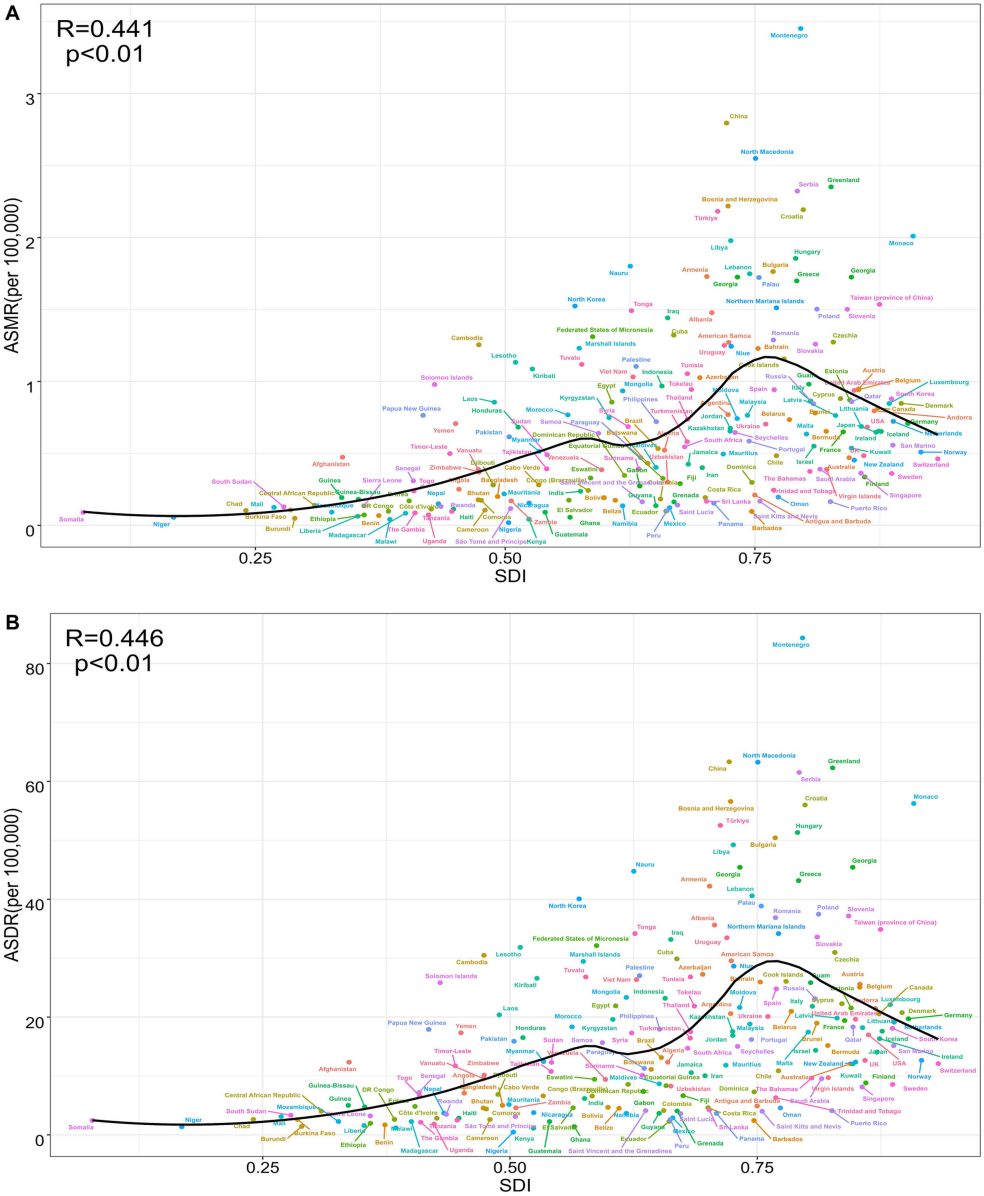
The correlation between TBL cancer attributable to SHS exposure in ASMR and SDI (R = 0.441, *p* < 0.01) **(A)**, between TBL cancer attributable to SHS exposure in ASDR and SDI (R = 0.446, *p* < 0.01) **(B)**.

A national-level analysis in 2021 revealed a significant positive correlation between the ASMR of TBL cancer attributable to SHS exposure and the SDI (R = 0.441, *p* < 0.01) (Figure 3A). This relationship exhibited a distinct non-linear pattern: when SDI < 0.75, ASMR gradually increased with rising SDI; however, when SDI > 0.75, ASMR began to decline, forming an inverted U-shaped distribution. Significant geographical disparities were evident in the regional distribution. Several countries deviated notably from expected ASMR levels. Montenegro, China, North Macedonia, and Greenland exhibited substantially higher ASMR than anticipated based on their SDI. At the same time, many African countries — including Nigeria, Malawi, Kenya, and Burundi — demonstrated significantly lower ASMR compared to nations with similar SDI levels (Figure 3A). Notably, several high-SDI countries such as Switzerland, Sweden, Finland, and Singapore maintained relatively low ASMR levels (Figure 3A). The ASDR displayed distribution patterns highly consistent with ASMR, showing a significant positive correlation with SDI (R = 0.446, *p* < 0.01) and exhibiting the same inverted U-shaped trend (Figure 3B). This consistency not only reinforces the reliability of the findings but also underscores the complex relationship between the TBL cancer burden attributable to SHS exposure and socioeconomic development levels.

### 3.5 APC analysis for TBL cancer burden attributable to SHS exposure

**Figure 4:**
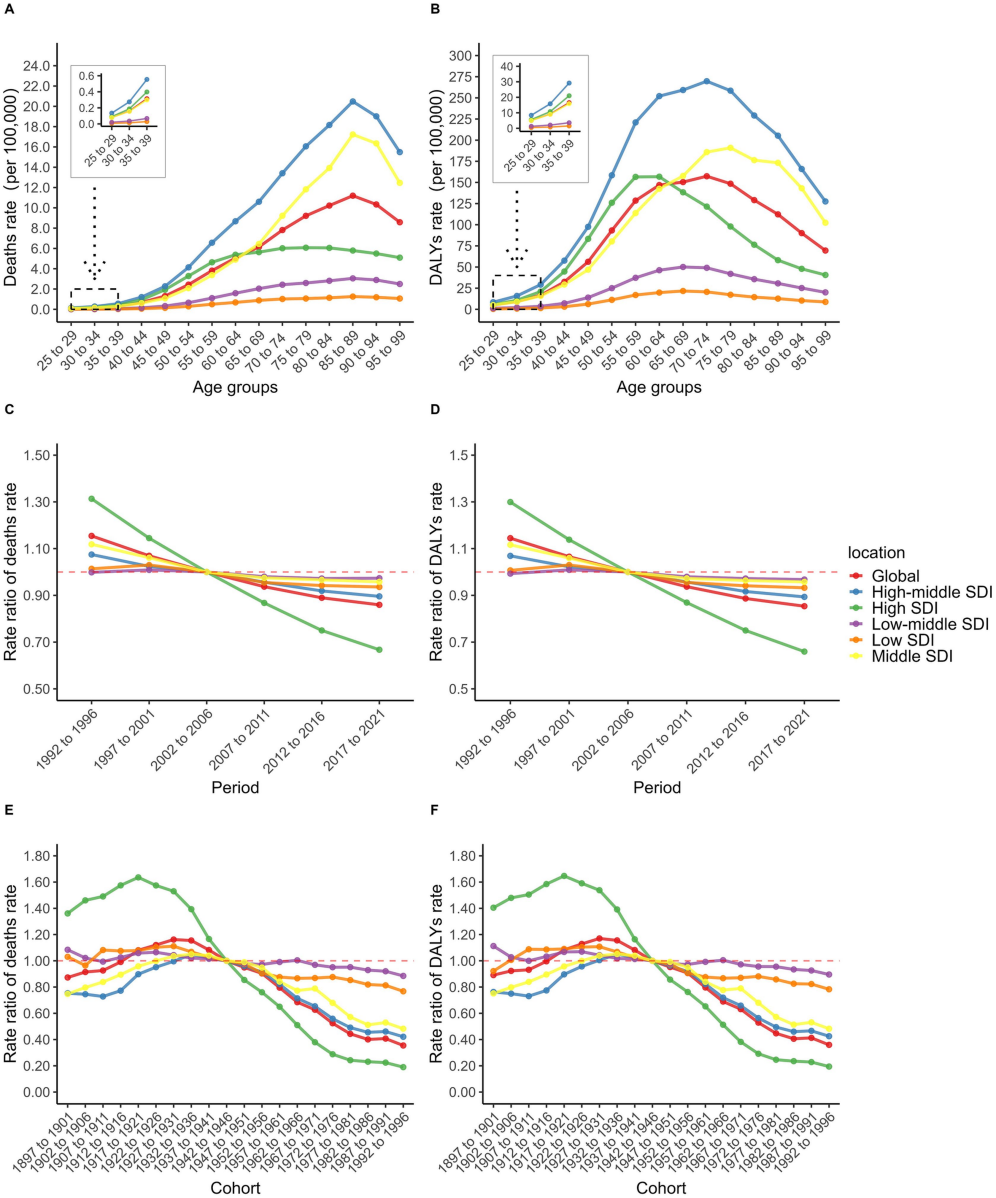
Age-specific TBL cancer deaths rate **(A)** and DALYs rate **(B)**; Period RR of TBL cancer deaths rate **(C)** and DALYs rate **(D)**; Cohort RR of TBL cancer deaths rate **(E)** and DALYs rate **(F)** attributable to SHS exposure.

The age effect revealed significant heterogeneity across different SDI regions. The high-middle SDI region exhibited the highest death rates across all age groups, with a linear increase from ages 25–29 to 85–89, followed by a gradual decline (Figure 4A). This may be due to the fact that these two regions experienced decades of high smoking rates and SHS exposure, and the implementation of relevant tobacco control policies lagged behind that of High SDI regions. The middle SDI region and the global average displayed similar inverted V-shaped distributions, peaking at ages 85–89 before decreasing (Figure 4A). The high SDI region showed a distinct pattern: death rates increased gradually from ages 25–29 to 65–69 (second only to the high-middle SDI region), then stabilized and declined below the global average (Figure 4A). Notably, in the middle SDI region, the death rate surpassed both the high SDI region and the global average at ages 65–69, rising to the second-highest level. In contrast, the low-middle and low SDI regions showed gradual increases in death rates from ages 25–29 to 75–79, followed by relative stability, with the disease burden consistently remaining below the global level (Figure 4A).

Sex-stratified analyses revealed notable differences, despite similar overall mortality patterns between males and females. In the high-middle SDI region, middle SDI region, and globally, male death rates peaked at ages 90–94 before declining rapidly (Supplementary Figure S5A). In contrast, female death rates peaked at 85–89, followed by an initial decline and then a secondary increase (Supplementary Figure S5B). Particularly in the high SDI region, male death rates remained relatively stable after ages 65–69, consistently staying below the global average (Supplementary Figure S5A). In comparison, female death rates began to fall below the global level from ages 55–59, displaying this trend approximately 10 years earlier than males (Supplementary Figure S5B). This may be due to female having less cumulative effects from SHS exposure than males.

Period effect analysis revealed an overall declining trend from 1992 to 2021. The high SDI region demonstrated the most substantial improvement, with the period rate ratio (RR) decreasing steadily from its peak in 1992–1996 to the lowest level in the most recent period (Figure 4C). Although other SDI regions also exhibited declining trends, their period RR values exceeded the global average after 2002–2006, particularly in the low-middle SDI region (Figure 4C). This region showed a progressive increase, rising from the lowest level in 1992–1996 to the highest level after 2002–2006 (Figure 4C). This may be related to the industrialization process and economic development. Notably, before 2002–2006, the high SDI region had the highest period RR among males (Supplementary Figure S5C), while the middle SDI region exhibited the highest period RR among females (Supplementary Figure S5D). After 2002–2006, period RR values in all SDI regions—except the high SDI region—exceeded the global average among females (Supplementary Figure S5D).

Cohort effect analysis showed that in the high SDI region, the cohort RR increased slightly between the 1897–1901 and 1917–1921 birth cohorts before declining, then decreased consistently from the highest to the lowest level after the 1942–1946 cohort (Figure 4E). This could be related to the development of medical and the improvement of people’s health awareness. The middle SDI and high-middle SDI regions, along with the global average, followed a similar trend, with brief increases from the 1897–1901 to 1932–1936 cohorts before gradually declining (Figure 4E). However, these regions maintained cohort RR values above the global average after 1942–1946 (Figure 4E). In contrast, the low-middle and low SDI regions showed minimal improvement in cohort RR, eventually surpassing other regions and the global average after 1942–1946 (Figure 4E). Sex disparities were most evident before the 1942–1946 cohort. Among males, the middle SDI region recorded the lowest cohort RR (Supplementary Figure S5E), while among females, the high-middle SDI region had the lowest (Supplementary Figure S5F). Importantly, the burden of DALYs from TBL cancer attributable to SHS exposure exhibited similar patterns to death rates across age, period, and cohort effects (Supplementary Figure S6).

### 3.6 ASMR projection for TBL cancer attributable to SHS exposure from 2022 to 2046

**Figure 5:**
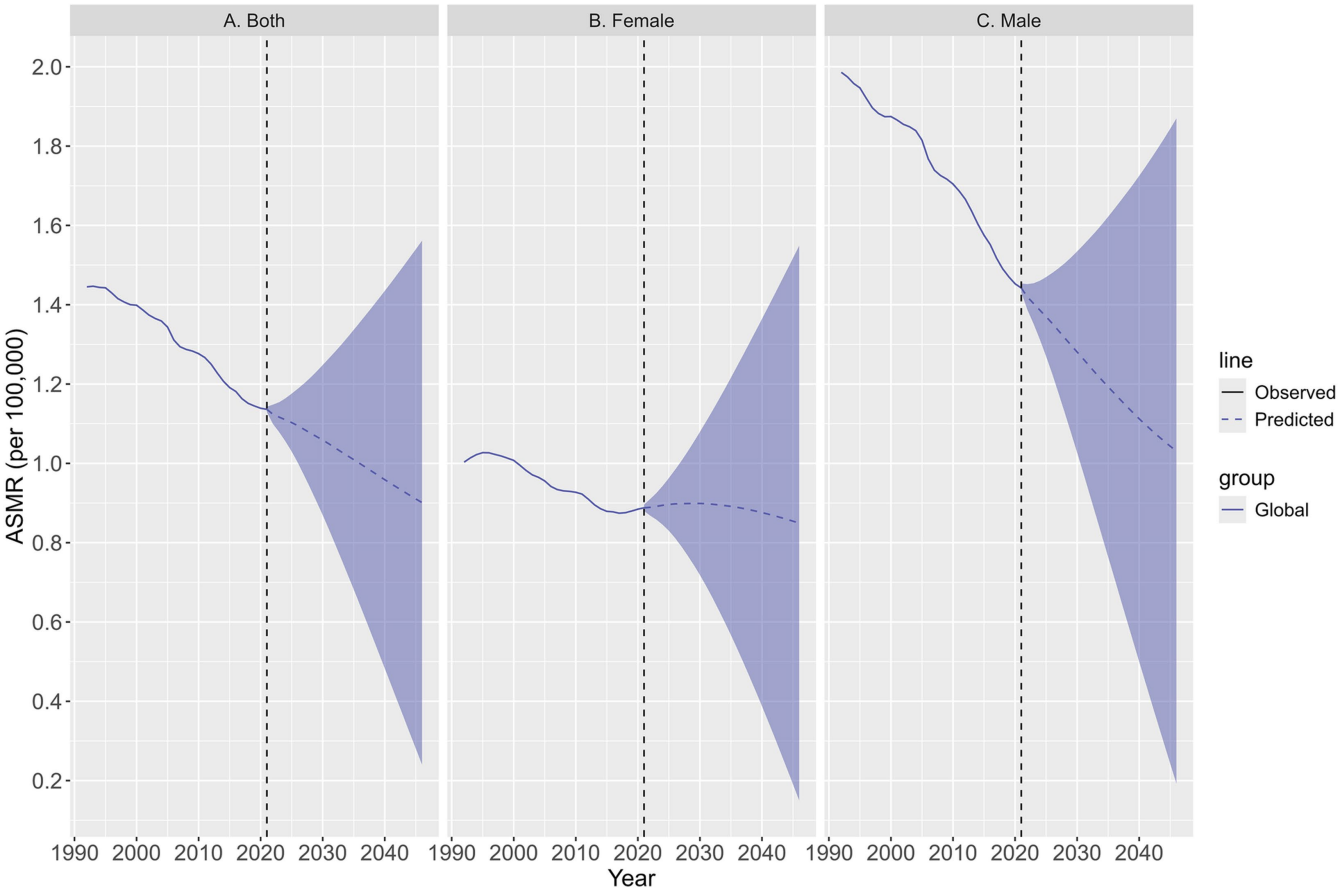
The temporal trends of ASMR in TBL cancer for both sexes **(A)**, females **(B)**, males **(C)** attributable to SHS exposure from 1992 to 2046. The solid line represents the observational values, while the dashed line shows the predicted values.

The ASMR for TBL cancer attributable to SHS exposure is projected to decline gradually from 2022 to 2046, with notable differences between sexes. In 2022, the overall ASMR is estimated at 1.12 (95% UI: 1.10 to 1.15), with significantly higher ASMR in men than in women (Figure 5). By 2046, the overall ASMR is expected to further decline to 0.90 (95% UI: 0.24 to 1.56), with the male ASMR projected to decrease to 1.03 (95% UI: 0.19 to 1.87) and the female ASMR is expected to decline to 0.85 (95% UI: 0.15 to 1.55) (Figure 5).

## 4 Discussion

Based on the GBD data, this research systematically evaluated the global disease burden of TBL cancer attributable to SHS exposure from 1992 to 2021. Through stratified analyses, we comprehensively investigated the evolving characteristics of this public health issue across multiple dimensions, including geographic regions, national development levels, and demographic variables (sex and age). The findings revealed that over the past 30 years, while the absolute number of deaths and DALYs due to SHS-attributable TBL cancer increased to varying degrees worldwide, both the ASMR and ASDR exhibited declining trends. These trends may be linked to global population growth and aging (31).

When examining disease burden trends from 1992 to 2021, marked heterogeneity was observed across SDI regions. The high SDI region achieved the most substantial progress over this period, with significant reductions in both absolute deaths and DALYs, as well as the fastest declines in ASMR and ASDR. This significant improvement is primarily attributed to several key factors: the implementation of comprehensive tobacco control programs (32–34), the widespread adoption of low-dose computed tomography (LDCT) screening (35) and increased health awareness (36). LDCT screening for individuals with long-term exposure to SHS environments can detect TBL cancer early and reduce TBL cancer mortality rates (37–40). The current increase in health awareness has also prompted people to advocate for the implementation of smoking cessation legislation (41–43). However, improvements in ASMR were relatively limited in low-middle and low SDI regions. Although some developing countries have begun to recognize the harmful risks of SHS exposure (44), these countries and regions lack the necessary resources for advanced diagnostic and treatment methods, and must balance multiple health threats within limited public health resources, making it difficult for TBL cancer prevention and treatment to receive adequate attention (45). Period and cohort effects further underscored the dynamic differences among SDI regions. In high SDI regions, early economic development was associated with increased smoking rates and limited awareness of SHS hazards, resulting in higher SHS exposure among earlier birth cohorts. However, in more recent periods, the intensity of SHS exposure and the associated TBL cancer burden declined due to improved health awareness, better access to healthcare, the implementation of smoking bans and cessation initiatives (27, 46, 47) and the implementation of the WHO Framework Convention on Tobacco Control (WHO FCTC). Conversely, later birth cohorts in low SDI regions may be experiencing increased SHS exposure due to industrialization, economic growth, rising tobacco use, and slower healthcare development (48–50). Notably, although current data indicate a relatively low disease burden in low SDI regions, this may be influenced by issues related to data availability and quality (51–53), necessitating cautious interpretation.

A national-level analysis in 2021 revealed that the top ten countries with the highest ASMR for TBL cancer attributable to SHS exposure were predominantly located in East Asia, Western Europe, and North America. This geographic distribution closely aligns with the historical patterns of tobacco use in these regions (45), reflecting the cumulative effects of long-term SHS exposure. Among them, European countries account for half, a phenomenon that deserves particular attention. Although both global ASMR and ASDR have shown declining trends over the past 30 years, indicating that the WHO Framework Convention on Tobacco Control (WHO FCTC) has made significant progress in reducing tobacco use since its implementation in 2003, Europe’s deeply entrenched social smoking culture continues to hinder the effective control of SHS exposure. In particular, the high social acceptance of smoking in public and social settings contributes to persistent SHS exposure, thereby exacerbating the population-level burden of TBL cancer (54). China, as the world’s largest consumer of tobacco (8), ranks second globally in ASMR for TBL cancer attributable to SHS exposure. Although China has ratified the WHO FCTC, it has yet to enact national smoke-free legislation prohibiting smoking in public places and workplaces (55). Previous research has shown that over 70% of adult males and 50 to 60% of adult females in China are exposed to SHS in the home and workplace environments (56). Therefore, multidimensional intervention strategies are urgently needed to reduce the disease burden of TBL cancer. In addition to enhancing smoking cessation services and improving healthcare accessibility, raising public awareness about the health hazards of SHS is crucial. Meta-analyses have shown that lifelong non-smokers living with smokers face a 20 to 30% higher risk of developing TBL cancer compared to those without SHS exposure—a finding that warrants serious attention (57, 58).

Stratified analysis by sex revealed significant sex disparities in the disease burden of TBL cancer attributable to SHS exposure. This disparity can be interpreted through three key dimensions. First, it may relate to differences in smoking prevalence, with higher smoking rates among males resulting in greater SHS exposure for men in both social and occupational settings. Second, although females generally have lower smoking rates, their proportion as passive smokers is substantial. Global data indicate that 35% of non-smoking women are regularly exposed to SHS (51), particularly in domestic settings (59). This pattern of chronic, low-dose exposure may explain why the SHS-related TBL cancer burden remains significant among females despite their lower active smoking rates. Third, women may exhibit heightened biological sensitivity to certain tobacco-related carcinogens, potentially due to a greater susceptibility to p53 and K-RAS gene mutations, as well as interactions between tobacco carcinogens and estrogen (14, 60). These molecular-level differences may help explain why females could face elevated carcinogenic risks even at comparable exposure levels. Nevertheless, in terms of observed disease burden, males demonstrate significantly higher levels than females, both in absolute numbers and age-standardized rates.

This study revealed a significant non-linear association between the disease burden of TBL cancer attributable to SHS exposure and the SDI, demonstrating a characteristic inverse U-shaped relationship. This pattern may be explained by the similarly shaped relationship between smoking prevalence and socioeconomic development (61). At the low SDI stage, limited economic development restricts access to tobacco products, resulting in lower smoking prevalence and, consequently, relatively low levels of SHS exposure within the population. In the middle SDI stage, rapid economic growth is often accompanied by a substantial rise in tobacco consumption. However, due to underdeveloped tobacco control policies and lagging public health regulations, the risks of SHS exposure increase significantly. Finally, at the high SDI stage, greater awareness of the health hazards of smoking and SHS exposure, combined with improved public health consciousness and the implementation of stricter tobacco control measures, leads to a decline in the disease burden. Based on these findings, countries at the middle SDI level should prioritize strengthening health education and implementing tobacco taxation and control policies to help reduce the burden of SHS-attributable TBL cancer.

Predictive modeling indicates a declining trend in the ASMR for TBL cancer attributable to SHS exposure. This decline is largely due to the implementation of multi-level tobacco control interventions worldwide, including tobacco taxation, packaging regulations, and anti-tobacco media campaigns (62–64). However, with approximately 37% of the global population still exposed to SHS environments (65), and this study reporting 97,910 SHS-attributable TBL cancer deaths and 2,355,866 DALYs in 2021, SHS exposure remains a critical public health challenge that requires continued and targeted intervention.

Four key recommendations emerge for addressing this issue. First, for middle SDI regions, strengthening smoke-free environment policies: bans on indoor and workplace smoking can significantly reduce SHS exposure, with particular emphasis on indoor smoking restrictions, as indoor exposure rates often exceed workplace exposure due to the amount of time people spend indoors. Second, optimizing TBL cancer screening strategies: incorporating SHS exposure into screening criteria could enhance early detection rates, though implementation should be region-specific. High SDI regions could integrate SHS exposure into existing low-dose computed tomography (LDCT) screening programs; middle SDI regions might adopt stratified screening approaches prioritizing high-risk populations; and low SDI regions, given resource constraints, should prioritize cost-effective preventive measures such as robust tobacco control programs or improved indoor ventilation systems to reduce SHS exposure. Third, for low SDI regions, enhancing risk communication: increasing public awareness about the health risks of SHS exposure, especially concerning vulnerable groups such as pregnant women and children (66–69), is essential for promoting health-conscious behaviors and reducing long-term disease burden. Fourth, we recommend adopting the theoretical framework of a “life-course strategy” (70). During childhood and adolescence, emphasis should be placed on promoting awareness of the harms of SHS exposure and fostering healthy behavioral consciousness. During the middle-aged population stage, tobacco control management in workplaces and public environments should be strengthened. In the older adult stage, TBL screening and health monitoring should be enhanced.

This study is the first to describe and explore long-term trends in the disease burden of TBL cancer attributable to SHS by age, gender, location, and SDI, utilizing APC and BAPC models. It fills an important research gap in this field, which represents a unique contribution of our study. However, several limitations should be acknowledged. First, the accuracy and robustness of GBD estimates heavily depend on the quality and completeness of input data used for modeling. GBD2021 did not include TBL data from SHS exposure for populations under 25 years of age, and the indicators for SHS exposure do not include new tobacco products such as e-cigarettes or secondhand aerosols (71). This may have led us to underestimate the disease burden trends of TBL caused by SHS exposure, and future research should further incorporate monitoring and assessment of such exposures. Moreover, in low SDI regions and sparsely populated countries, systematic underreporting may occur due to inadequate disease surveillance and vital registration systems. Consequently, trend analyses for these regions should be interpreted with caution and validated by future research. Second, our research findings are based on ecological data. In analyzing the non-linear association between TBL cancer burden attributable to SHS exposure and the SDI, we calculated simple linear Pearson correlation coefficients, which may not accurately reflect the true strength of non-linear relationships. Moreover, we did not employ multivariable ecological models to adjust for other potential confounding factors (such as indoor air pollution, dietary factors, genetic factors, etc.) and measurement heterogeneity that could impact TBL cancer outcomes. Furthermore, our predictions of disease burden trends are based on the assumption that risk factor exposure trends remain unchanged; however, this assumption may no longer hold if new tobacco control measures are introduced. We anticipate that these issues can be addressed in future research. Finally, the lag effect of interventions may influence our assessment of the effectiveness of current prevention and control measures. Although many countries have implemented tobacco control policies, the full population-level health benefits of these interventions may take time to be reflected in disease burden data. Despite these limitations, a key strength of the GBD study framework lies in its regular update mechanism. With ongoing improvements in data availability and methodology, future estimates will more accurately capture dynamic changes in the global burden of SHS-related TBL cancer. We look forward to future studies that conduct comparative risk assessments between SHS and other TBL risk factors (such as indoor air pollution, outdoor particulate matter, etc.), or evaluate the cost-effectiveness of specific SHS intervention measures under different environmental conditions.

## Data Availability

The original contributions presented in the study are included in the article/Supplementary material, further inquiries can be directed to the corresponding author.

## Footnotes

1
http://ghdx.healthdata.org/gbd-results-tool
