# Supplementary material for: Global burden of tracheal, bronchus, and lung cancer attributable to second-hand smoke exposure from 1992 to 2021: an age-period-cohort analysis and 25-year mortality projections
Source: Front Public Health. 2025 Nov 25;13:1625876. doi: 10.3389/fpubh.2025.1625876 (PMC12685916; doi:10.3389/fpubh.2025.1625876)

Figure S1: The spatial distribution of TBL cancer ASMR (A) and ASDR (B) for males attributable to SHS exposure in 2021 and TBL cancer ASMR (C) and ASDR (D) for females attributable to SHS exposure in 2021.


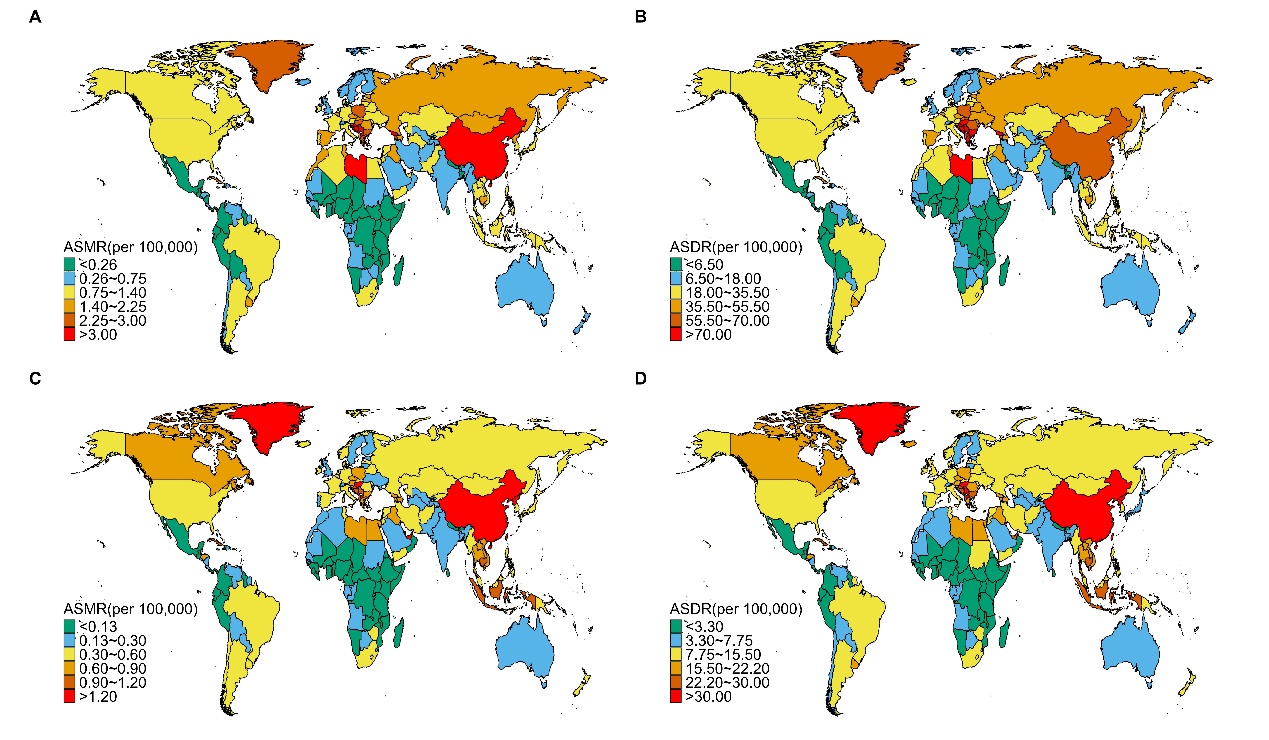


Figure S2: The spatial distribution of EAPC in ASMR (A) and ASDR (B) of TBL cancer for males attributable to SHS exposure and EAPC in ASMR (C) and ASDR (D) of TBL cancer for females attributable to SHS exposure from 1992 to 2021.


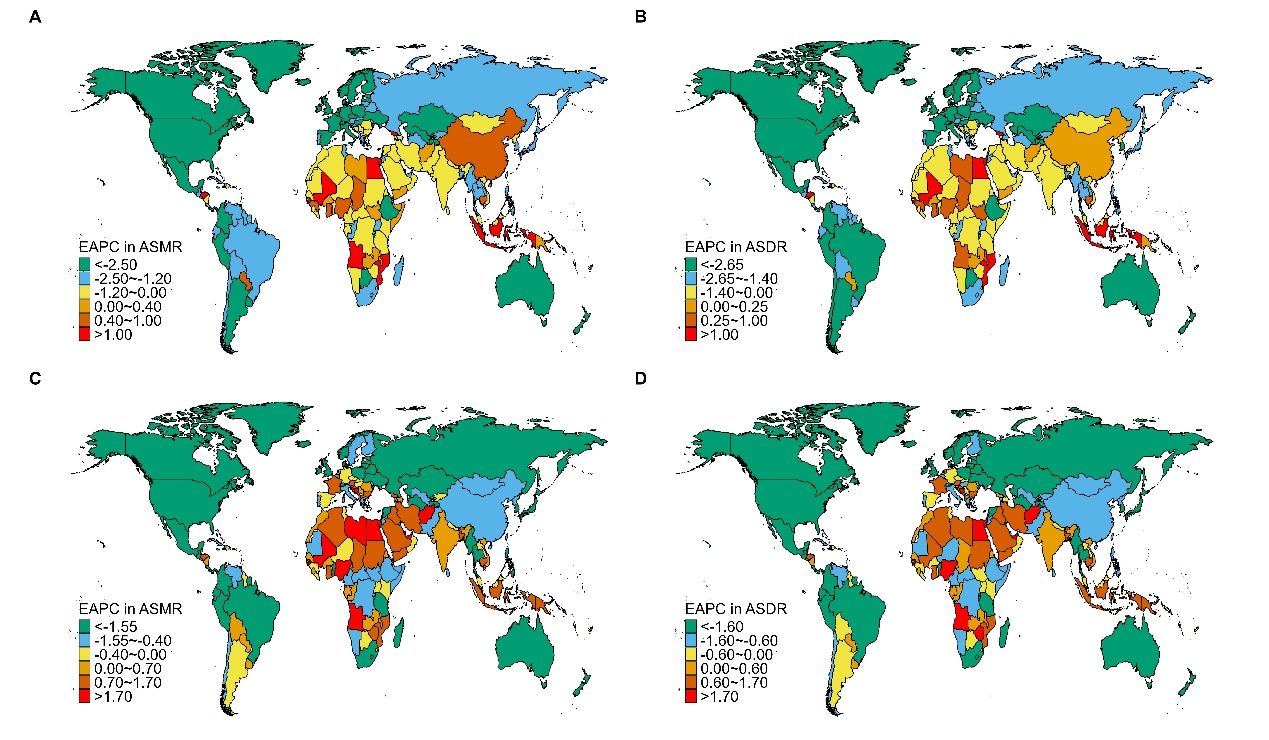

Supplement: Supplementary file 4 [file Supplementary_file_1.docx]
