# Supplementary material for: Global burden of tracheal, bronchus, and lung cancer attributable to second-hand smoke exposure from 1992 to 2021: an age-period-cohort analysis and 25-year mortality projections
Source: Front Public Health. 2025 Nov 25;13:1625876. doi: 10.3389/fpubh.2025.1625876 (PMC12685916; doi:10.3389/fpubh.2025.1625876)
Supplement: Supplementary file 5 [file Supplementary_file_2.docx]

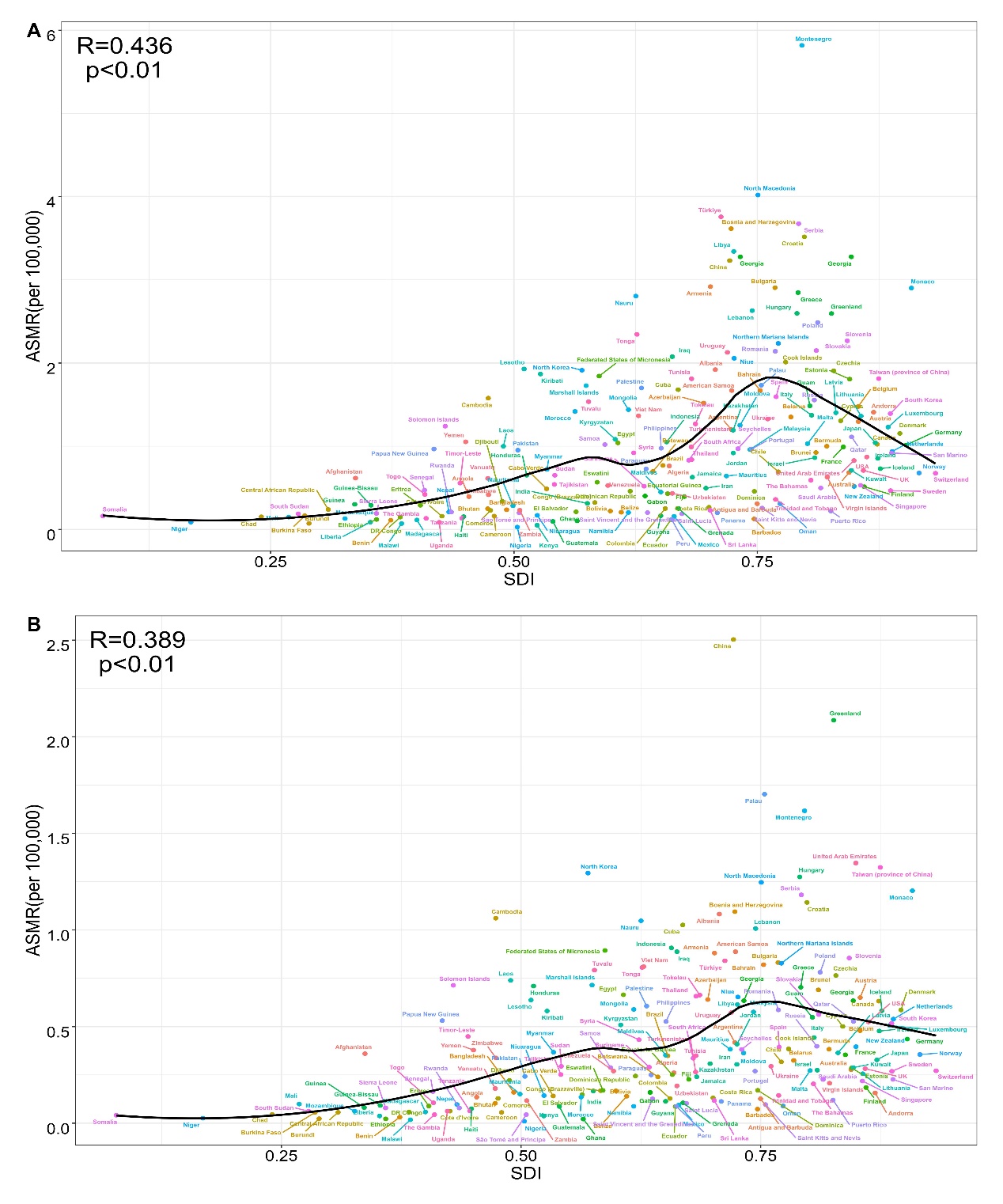


Figure S3: The correlation between ASMR of TBL cancer attributable to SHS exposure and SDI for males (R=0.436, p<0.01) (A), between ASMR of TBL cancer attributable to SHS exposure and SDI for females (R=0.389, p<0.01) (B).


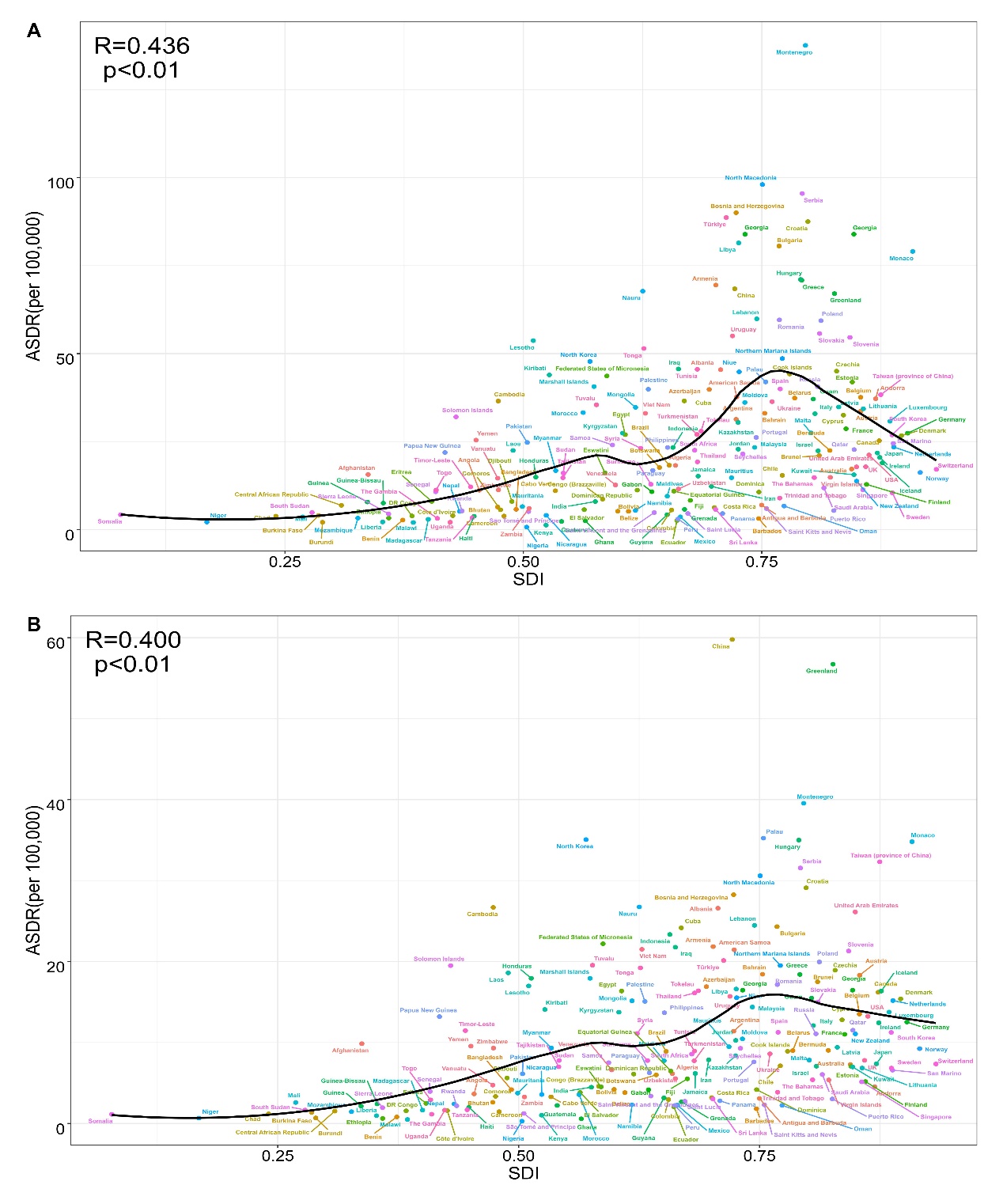


Figure S4: The correlation between ASDR of TBL cancer attributable to SHS exposure and SDI for males (R=0.436, p<0.01) (A), between ASDR of TBL cancer attributable to SHS exposure and SDI for females (R=0.400, p<0.01) (B)
