# Supplementary material for: Global burden of tracheal, bronchus, and lung cancer attributable to second-hand smoke exposure from 1992 to 2021: an age-period-cohort analysis and 25-year mortality projections
Source: Front Public Health. 2025 Nov 25;13:1625876. doi: 10.3389/fpubh.2025.1625876 (PMC12685916; doi:10.3389/fpubh.2025.1625876)
Supplement: Supplementary file 6 [file Supplementary_file_3.docx]

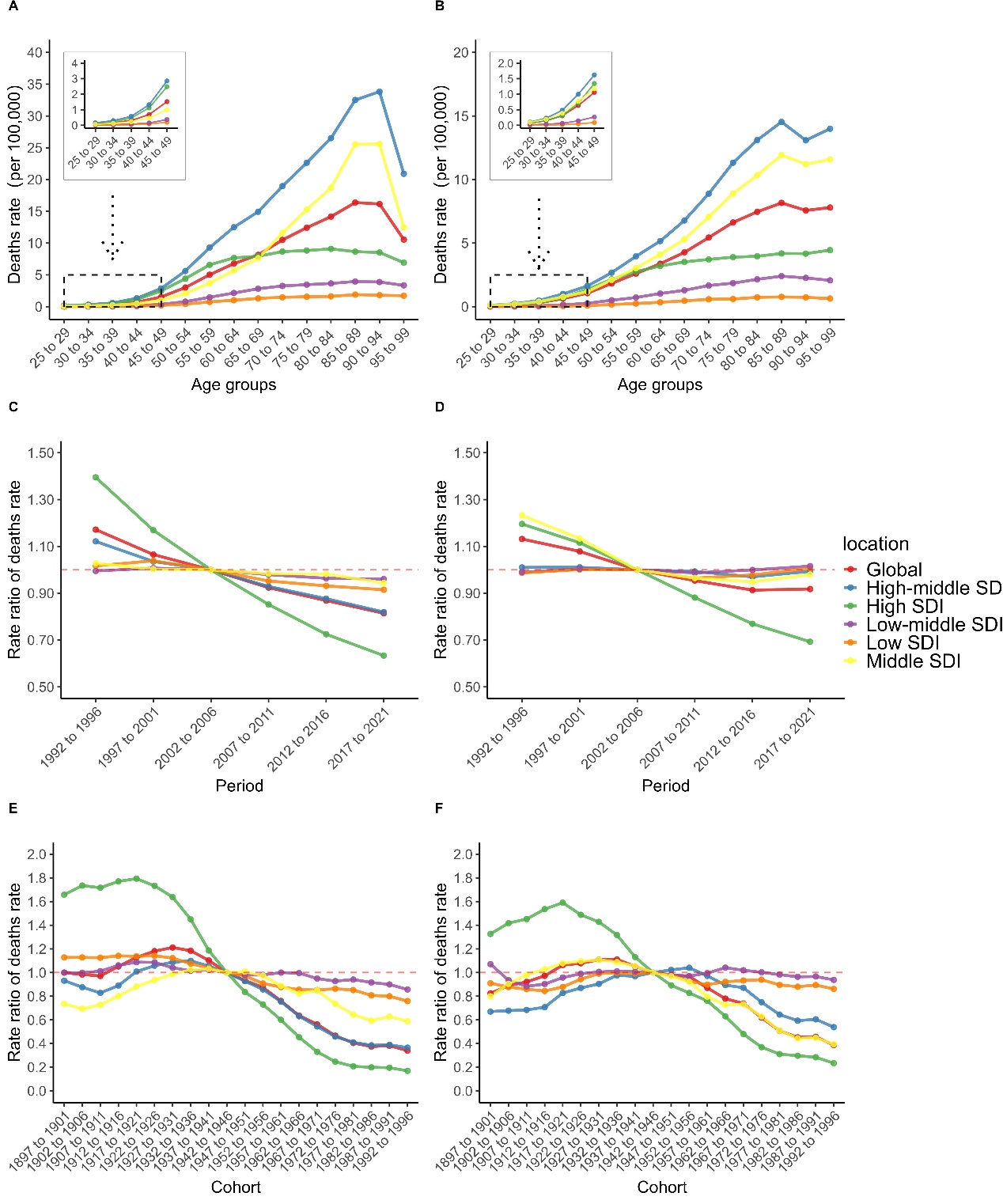


Figure S5: Age-specific TBL cancer deaths rate for males (A) and females (B); Period RR of TBL cancer deaths rate for males (C) and females (D); Cohort RR of TBL cancer deaths rate for males (E) and females (F) attributable to SHS exposure.


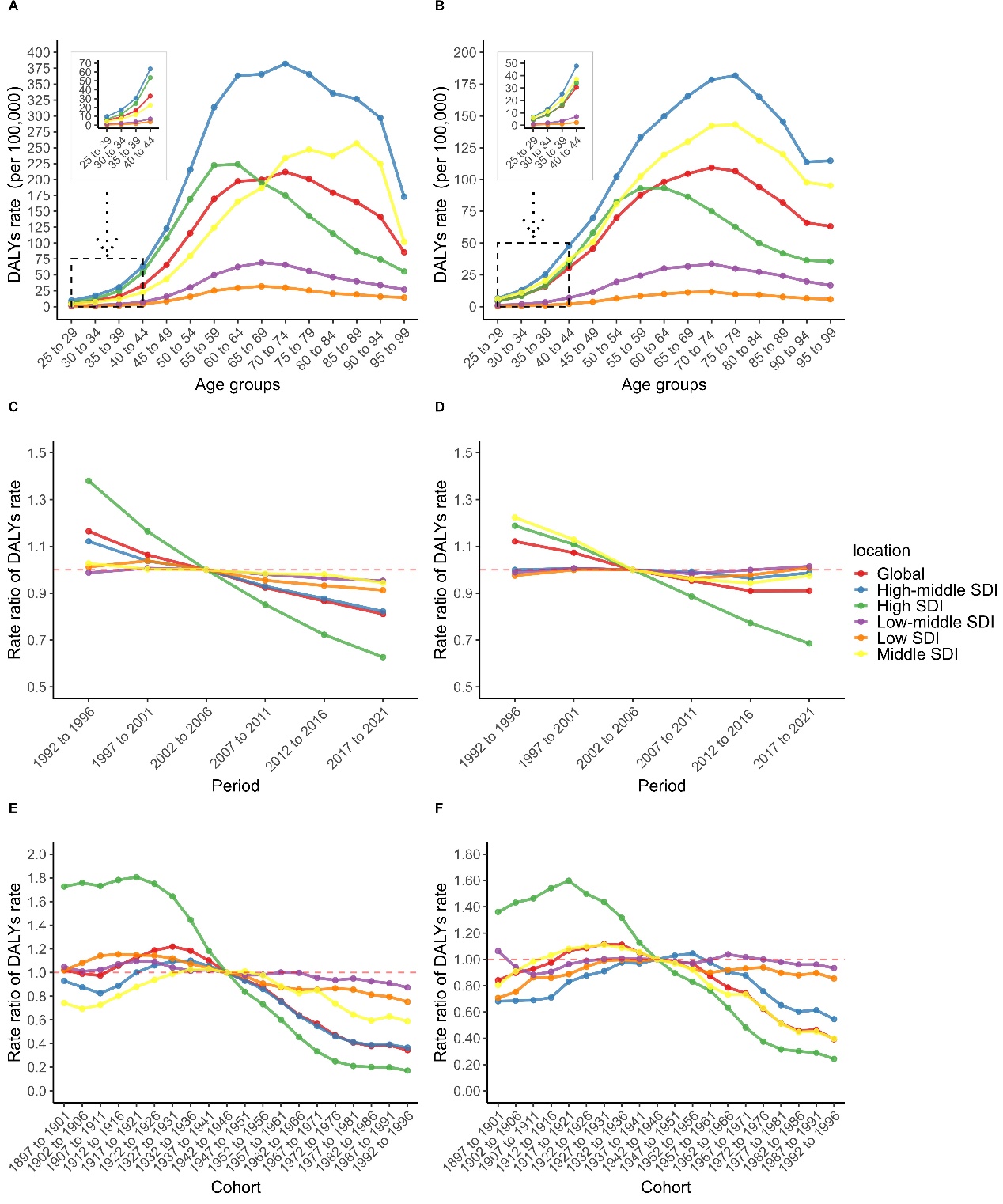


Figure S6: Age-specific TBL cancer DALYs rate for males (A) and females (B); Period RR of TBL cancer DALYs rate for males (C) and females (D); Cohort RR of TBL cancer DALYs rate for males (E) and females (F) attributable to SHS exposure.
